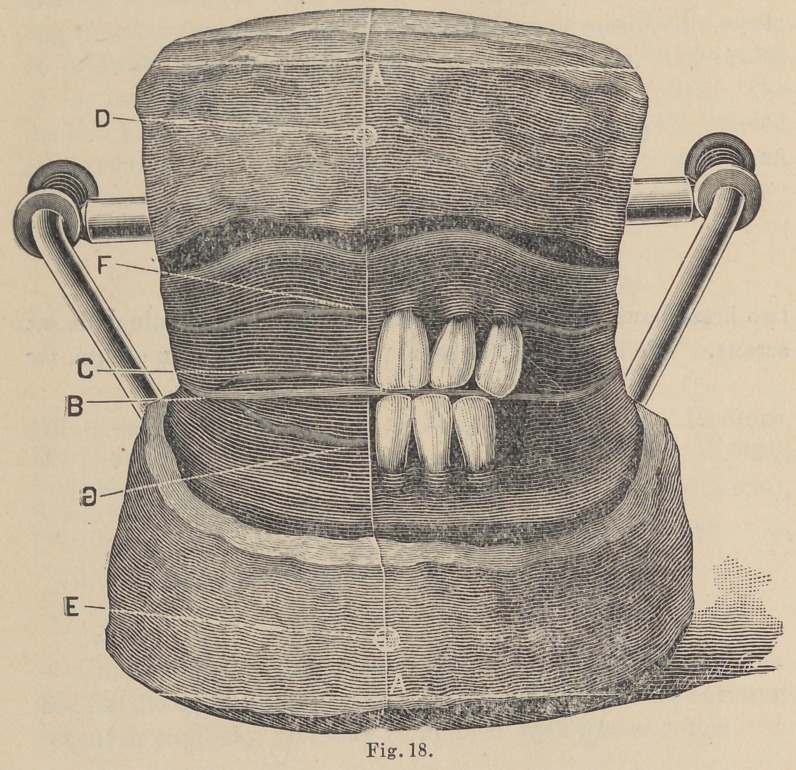# Articulation of Artificial Teeth

**Published:** 1894-06

**Authors:** Grant Molyneaux

**Affiliations:** Cincinnati, O.


### Articulation of Artificial Teeth.

(*Continued from Page* 142.)

BY GRANT MOLYNEAUX, D.D.S., CINCINNATI, O.

NOTE.—Figs. 1 to 5, Page 133, were inverted by printer and should be observed with book upside down.

Some criticism might be made for not reproducing the engraving taken from Dr. Black's Anatomy perfectly, but we only desired to show the relation of the teeth, especially the cutting edges, consequently the other part of drawing was sacrificed. The drawings were only used by us to save time, as we have a skull with an arrangement of teeth, and if photographed would hardly be distinguishable from Dr. Black's drawings.

The "Anatomical Articulator" is composed of brass wire and tubing and illustrated in Fig. 10. It consists of a base and

Fig. 10.

two brass bows, the bows being detachable by loosening the set screws. The articulator as seen in cut is in position of use, *the*

Fig. 13.

*narrow bow being uppermost at all times and upon which the upper model, whether that being an articulating model or a model upon which a set of teeth is to be arranged, is to be mounted; contra, the lower model which is always to be mounted on the wide bow.*

The cross bar or tube to which the narrow bow of the articulator is attached, corresponds to the base of our triangle or the line from *a* to *a* Fig. 9 and *c* to *c* Fig. 16.

At either extremity of the cross-bar is an eyelet through which the "condyle" of the articulator works.

The two spiral springs back of the condyle take the place of the muscles and serve to keep the models in the position of occlusion, except when moved about to get the different bearings of the artificial teeth. With a normal natural denture the opposing

Fig. 18.

teeth are in positive contact during occlusion and the same condition should be carried out with an artificial denture. Before attempting this plan of articulating teeth, the conditions must be made favorable to successful results.

1st. We must obtain a correct occlusion of the lower jaw.

2d. The wax articulating models or "trial plates" should represent the exact length of lips. (Fig. 18.)

3d. The patient must be observed full face and profile, and facial contour perfectly established.

4th. The median line of face marked distinctly on the trial plates.

We will now try to give accurately the steps as they are followed in using this machine for the articulation of artificial teeth. The two set screws are loosened and the brass bows pushed firmly back into the sockets and then tightened. The occluding wax trial plates should represent the exact exposure of teeth (Fig. 18, F to B, G to B, and also the plane of the cutting edges of the incisors, Fig. 18 B.) *The horizontal surface of the base of the plaster models should be trimmed to represent the same plane as the occluding surface of the wax articulating models*, Fig. 18 A and A. The purpose of this is to mount the case as squarely as possible in the articulator.

The lower model is adjusted upon the wide bow, the heels pointing towards the condyles, the "bite" wax and upper model also in position, when the narrow bow is turned to rest on the base of upper model. We now take a pair of dividers, separated to measure four inches, placing one foot in a small depression at the condyle and measuring the distance to median line of wax bite from each condyle.

Rule: Let the median line of the mouth, F to T Fig. 9, fall midway between the condyles of the articulator and at the same time let the median line of the bite at the occluding edge of wax be equi-distant (four inches) from each condyle; when in this position pour plaster, quite thin, over the narrow bow and upper model and at once lift the articulator up by the base and make a bed of plaster on table into which the wide bow and lower model is placed. When the plaster hardens, the excess should be trimmed away and we are ready for the teeth.*

We must now repeat a previous statement. The wax articulating models or "bite" should represent the exact length of lips, the proper contour of lips and the median line of face. If these points have been faithfully observed then the lower wax articulating model at the median line would represent exactly the position of the central incisor teeth, inferior. All that is necessary is to cut down to the base plate through the median line of the wax and take out a section, upper and lower, *on one side only*, and enough to admit of placing the central, lateral and cuspid teeth on one side (Fig. 18.) After these teeth are appproximately placed, the measurements given in last paper are to be followed to fix their exact position, when they should be waxed fast. We have stated that the wax models represent the length of lips, which is true, but we have not accounted for the overlap. When the overlap is determined, just that much should be removed from the occluding surface of the upper wax model. (Fig. 18, B to C.) After arranging the central, lateral and cuspid, enough wax is removed to admit of the bicuspids and lastly the molars. By this plan the trial plates will at all times be held firmly in contact with the plaster models. When we have one side complete, we begin on the opposite side and finish as the first.

It has been suggested by those not familiar with the articulator that a screw is necessary for opening and closing the bite. Such a screw is not only unnecessary but would be a detriment to the instrument. The wax establishes the bite at all times, and if any lengthening is necessary more wax can be added, if shortening is necessary some wax can be removed.

A word regarding the position of molar teeth may not be out of place. A custom prevalent with certain operators is placing the molar teeth too near the cheek. If a plumb line were dropped from the lingual border of the lingual cusp of the second in-

* For mounting a full upper or lower case where we have the natural teeth in one or the other jaws—obtain a model of the teeth and place this model in indentations on the wax trial plate and mount, making the same measurements as with the entire denture.

ferior molar, it would fall clear of the body of the jaw and not through the center of the edentulous ridge, as many artificial teeth are arranged. When the teeth are placed too near the cheek, it is constantly irritated and the teeth are, during lateral movement, thrown out of line of use. The position of the molar teeth would be more nearly correct if a perpendicular line falling through the buccal cusps would also fall through the center of the edentulous ridge. This arrangement might, upon first observation, appear to impede the movement of the tongue. Such is not the case, but to the contrary, the molar teeth are in the best position to receive the greatest assistance from the tongue and cheek in keeping the food in position for mastication.

In the selection of teeth, one should keep the requirements before him constantly, and select those teeth that will require the least alteration. There are very few sets of teeth, however, that can not be improved, for articulation, by judicious grinding. By referring to Fig. 12 or 3 and 13, the relative size of the cusps of bicuspids and molars will be observed. The buccal cusps of the superior teeth being longer than the lingual, and *vice versa* for the inferior.

These diagrams will indicate the necessity and method of grinding. For fear these drawings may be thought ideal, we take the liberty of reproducing another cut from Dr. Black's Anatomy, Fig. 13. This drawing being taken from life, is a most excellent subject for study, it indicating not only the plan by which artificial teeth should be articulated, but is right in line with our remarks on the angles of teeth. Compare with Fig. 3. Refer now to Fig. 16 and we observe that the first superior bicuspid tooth is the larger. In the lower denture the first is the smaller. In most artificial sets of teeth we find the first bicuspid above the smaller, and altogether too small to look well next to the cuspid. By reversing the order, placing the second artificial bicuspid for the first, or the larger of the bicuspids first, the appearance is not only improved but a better articulation is obtained.

There is one other mistake in many artificial sets of teeth, viz., the first inferior bicuspid lingual cusp is quite as large as the lingual cusp of the second bicuspid. Nature only intended one cusp (buccal) for these teeth, as a lingual cusp is not only useless, but it is in the way of the tongue. Remove it entirely with the stone. The manufacturers of artificial teeth can not construct moulds to suit every individual whim, but we believe, however, that they are trying to produce teeth as nearly life-like as possible, in both color and shape, and with a fair variety of moulds from either the standard makers, forms can be found that will suit the most fastidious. What little there is wanting we can usually add with our mineral paints and the grindstone.

With the above remarks and with the aid of Figs. 17, 16 and 13, we believe that the clew to a natural arrangement of the artificial teeth is clear, only needing a little practice to execute it rapidly. When one is familiar with this method the work can be performed in quite the same time as by the old methods, and with an infinitely greater degree of accuracy. The articulator has many other virtues, but I will refer to only two more. To those doing any amount of regulating, the instrument becomes invaluable, from the fact that the models can be mounted and studied as accurately as in the mouth.

To those doing any quantity of gold plate of continuous gum work, an enormous amount of labor can be avoided by taking the measurements of the completed dentures with a pair of dividers and marking them on the base of the models, together with the color and size of a central incisor. The set screws can then be loosened and the models with the bows intact laid away and properly labeled for future reference. They can be referred to at any time, and should the patient be a thousand miles distant duplicate dentures can be constructed and forwarded with every assurance of a successful result. One " base" is all that is really necessary; the bows can be made *ad libitum*, and when one case is mounted and the plaster hard, the models can be slipped out and a new set of bows adjusted for the next case.

We regret our inability to do the subject justice on paper, but if we have awakened in any person a desire to familiarize himself with and apply the method to suffering humanity, our labors will have been fully rewarded.

By referring to Fig. 18, the reader will observe the plan of arranging the wax articulating models and how the markings are made on the wax. A—A represents the base of the plaster models trimmed to conform to the same plane as B or the cutting edge of wax model from cuspid to cuspid, which line indicates the plane of the incisor teeth. The distance from C to B is the length of over-bite, and as the wax models show the length of lips, it is evident that the wax model must be made shorter by B to C. The wax on the occluding surface of the upper wax model must be trimmed off to the line C. Before trimming off the wax it will be necessary to use the dividers in order to retain the exact length of teeth. Place one foot of dividers in a depression on model at D and the other at B and fasten.

The lower can be marked from E to B at same time, say this distance is an inch and a half from the depression at D to B and from E to B. Mark this on base of models. Should, for any reason, the wax models become destroyed, or your dividers be changed, they can be easily re-adjusted by referring to marks on base of models.

The line B is made to approximate in the wax the curves described by the cutting edges of the teeth. C is the point to which the lower lip is dropped and F the point to which the upper lip is raised in laughing. The lower teeth are in position, the upper only approximately. After our measurements are made on the occluding surfaces of wax we trim off the upper wax from B to C and let the teeth overlap.

In concluding this paper we must make mention of the fact that if the bite is taken wrong, this articulator nor any other will make it right. Time spent in taking a correct occlusion of jaws and other markings will never be spent in vain, as it saves an endless amount of trouble.

---

Mrs. Jane L. Fowle, of Dedham, Mass., has been awarded $450 by a Boston jury against a dentist who extracted a sound tooth instead of a decayed one.

## Figures and Tables

**Fig. 10. f1:**
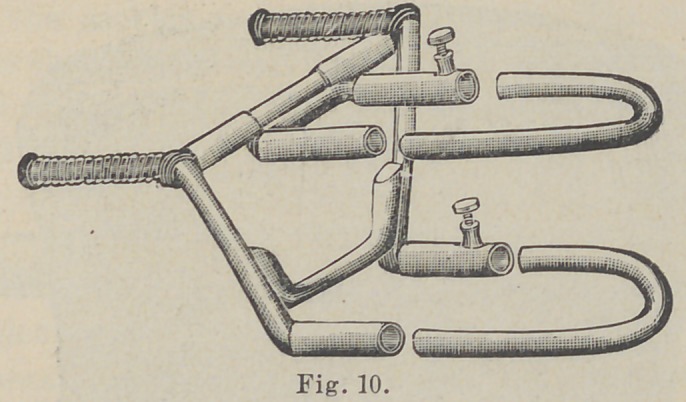


**Fig. 13. f2:**
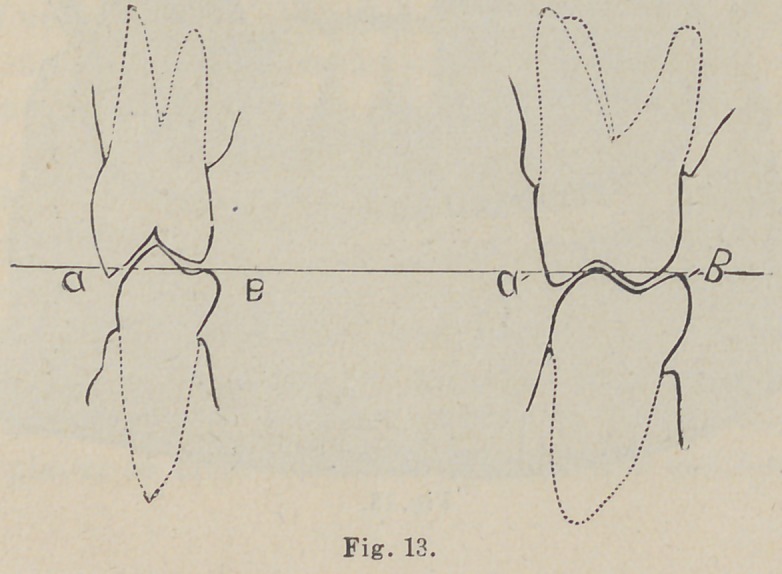


**Fig. 18. f3:**